# Development of a group-based diabetes education model for migrants with type 2 diabetes, living in Sweden

**DOI:** 10.1017/S1463423620000493

**Published:** 2020-11-09

**Authors:** Emina Hadziabdic, Sara Pettersson, Helén Marklund, Katarina Hjelm

**Affiliations:** 1 Department of Health and Life Sciences, Linnaeus University, Växjö, Sweden; 2 Department of Social and Welfare Studies, Linkoping University, Norrköping, Sweden; 3 Department of Public Health and Caring Sciences, Uppsala University, Uppsala, Sweden

**Keywords:** culturally appropriate health diabetes education model, migrants, type 2 diabetes

## Abstract

**Aim:**

To develop a diabetes education model based on individual beliefs, knowledge and risk awareness, aimed at migrants with type 2 diabetes, living in Sweden.

**Background:**

Type 2 diabetes is rapidly increasing globally, particularly affecting migrants living in developed countries. There is ongoing debate about what kind of teaching method gives the best result, but few studies have evaluated different methods for teaching migrants. Previous studies lack a theoretical base and do not proceed from the individuals’ own beliefs about health and illness, underpinned by their knowledge, guiding their health-related behaviour.

**Methods:**

A diabetes education model was developed to increase knowledge about diabetes and to influence self-care among migrants with type 2 diabetes. The model was based on literature review, on results from a previous study investigating knowledge about diabetes, on experience from studies of beliefs about health and illness, and on collaboration between researchers in diabetes care and migration and health and staff working in a multi-professional diabetes team.

**Findings:**

This is a culturally appropriate diabetes education model proceeding from individual beliefs about health and illness and knowledge, conducted in focus-group discussions in five sessions, led by a diabetes specialist nurse in collaboration with a multi-professional team, and completed within three months. The focus groups should include 4–5 persons and last for about 90 min, in the presence of an interpreter. A thematic interview guide should be used, with broad open-ended questions and descriptions of critical situations/health problems. Discussions of individual beliefs based on knowledge are encouraged. When needed, healthcare staff present at the session answer questions, add information and ensure that basic principles for diabetes care are covered. The diabetes education model is tailored to both individual and cultural aspects and can improve knowledge about type 2 diabetes, among migrants and thus increase self-care behaviour and improve health.

## Background and rationale for the development

Diabetes Mellitus (DM) is rapidly increasing globally, predominantly including type 2 diabetes (85%) and particularly affecting migrants living in developed countries (IDF, 2017; Cho *et al*., 2018). At the same time, this group shows difficulties in assimilating knowledge about diabetes and self-management through the education that is usually offered at diabetes clinics (Testa *et al*., 2015). The growing burden of type 2 diabetes among foreign-born persons represents a serious public health challenge for many European countries (Montesi *et al*., 2016). The ongoing migration is not expected to slow down and will generate increasing economic costs for several European national health systems (Montesi *et al*., 2016), and cause suffering for the individual (IDF, 2017).

Active participation in self-care, based on knowledge about the disease, is the most important cornerstone for a person’s self-management of type 2 diabetes (Testa *et al*., 2015; IDF, 2017; Socialstyrelsen, 2018; ADA, 2019). Thus, education should enhance a patient’s knowledge and skills regarding the management and empower them to take an active role in their treatment (Coppola *et al*., 2016; Chatterjee *et al*., 2018; ADA, 2019; Miller *et al*., 2020), aiming to achieve good glycaemic control to prevent diabetes-related complications (Testa *et al*., 2015; IDF, 2017; Socialstyrelsen, 2018). There is ongoing debate about what kind of teaching method gives the best result, but few studies have evaluated different methods for teaching migrant groups or ethnic minority groups (Hawthorne *et al*., 2008; Socialstyrelsen, 2012; 2018). The Diabetes Education and Self-Management for Ongoing and Newly Diagnosed (DESMOND) programme is a structured group-based educational programme for people with type 2 diabetes (run since 2008). It is used in the UK (Chatterjee *et al*., 2018) and Australia (Miller *et al*., 2020) and is one of the only programmes that have been extensively evaluated to show a variety of health outcome improvements. Some courses in the programme have been adapted for delivery to South Asian ethnic populations but the impact on ethnic minority groups needs to be evaluated (Chatterjee *et al*., 2018).

Previous qualitative studies, comparing foreign- and Swedish-born persons with type 2 diabetes living in Sweden, indicated dissimilarities, with foreign-born persons (migrants) having limited knowledge about diabetes and underestimating the seriousness of diabetes, compared to Swedes; this affected self-care behaviour (Hjelm *et al*., 1999; 2003; 2005; 2013). A previous study (Pettersson *et al*., 2018), measuring knowledge about diabetes, confirmed the hypothesis. In addition, the results showed that persons originating from non-European countries had the lowest level of knowledge about diabetes.

It is a challenge to understand the complex process of self-care and to develop appropriate education models that support patients in managing their chronic illness and maintaining or improving their health (ADA, 2019). Criticism of normal diabetes care, including education about diabetes, has emphasised the lack of patient involvement and the focus on control, instead of patient-centredness (Hörnsten and Graneheim, 2009). Education has been recognised as an essential aspect of care for diabetes by improving knowledge about the disease and thus enhancing the patient’s self-care and improving health outcomes (Coppola *et al*., 2016; Chatterjee *et al*., 2018; ADA, 2019; Miller *et al*., 2020). It is therefore important to examine what kind of education is most appropriate.

Normal care visits are often not culturally or individually adapted, in contrast to what is recommended (Socialstyrelsen, 2012; 2018). It is important to remember that one of the nurse’s main tasks is to support patients in self-care by teaching why assessment of knowledge about the disease is important (Leininger and McFarland, 2006). Thus, the implementation of interventions to improve knowledge about diabetes for persons with established diabetes should become a public health priority across the world (IDF, 2017).

Today, diabetes care in Sweden should be and is recommended to be based on the national guidelines for diabetes care (Socialstyrelsen, 2018). The majority of patients diagnosed with type 2 diabetes are managed in health care centres in primary health care, but patients with severe type 2 diabetes and with complications related to diabetes are managed by specialists at in-hospital diabetes clinics for adults. The ambition of Swedish diabetes care is to work in multi-professional teams (Socialstyrelsen, 2018). At the primary health care clinic, the patient should be managed by a team including a general practitioner (GP) particularly trained in diabetes management, a diabetes specialist nurse and if needed a podiatrist, a physiotherapist and a social worker. A GP and a diabetes specialist nurse should meet each patient at least once a year, and focus on quality of care documents, such as national guidelines and the national diabetes register. Other professionals should also meet the patient when there is a need. Together with the patient, targets for treatment are set, which are based on assessment of quality of life and the risk of complications. The aim is to achieve good results for the patient. It should be clarified that the patient must take great responsibility for self-care. Even though a multi-professional team is recommended, the reality often means that there is only a GP and a diabetes specialist nurse in the team, while the other professionals work on a consultancy basis (Socialstyrelsen, 2012; 2018).

Group-based diabetes education and culturally adapted diabetes education is recommended for persons diagnosed with type 2 diabetes, including migrants (SBU, 2009; Socialstyrelsen, 2012; 2018), but is scarce in Swedish diabetes care. Educational models are lacking.

Cultural tailoring is a concept that utilizes the understanding of the effects of cultural characteristics on health behaviours to design a useful intervention (Hawthorne *et al*., 2008; Attridge *et al*., 2014). Culturally appropriate health education can be defined as education that is tailored to the cultural and religious beliefs and linguistic skills of the targeted community, but also taking literacy skills into consideration (Attridge *et al*., 2014). Even though research activity in this field has increased over the last decade, showing that culturally appropriate diabetes education yields consistent benefits over conventional care in terms of improved glycaemic control and diabetes knowledge, there is a need for further studies to investigate successful aspects of culturally tailored education models for migrants with type 2 diabetes and to develop new models for diabetes education to be tested in order to determine their clinical significance (Hawthorne *et al*., 2008; Attridge *et al*., 2014; Chatterjee *et al*., 2018). Previous investigations focussing on culturally appropriate education (Hawthorne *et al*., 2008; Attridge *et al*., 2014; Creamer *et al*., 2016) have mainly focussed on ethnic minorities in the US, showing that many lack a theoretical base and a definition of the concept. They have been implemented with preplanned structured lectures, where the educator, usually a healthcare professional, teaches the patient about diabetes care, and does not proceed from the individuals’ own beliefs about health and illness, underpinned by their knowledge and guiding their health-related behaviour.

The aim is to develop a diabetes education model for migrants with type 2 diabetes living in Sweden, based on individual beliefs, knowledge and risk awareness.

## Development of the model

This article describes the development of a diabetes education model for migrants aimed to be used in an intervention study to increase knowledge about diabetes and thus influence self-care among migrants with type 2 diabetes. In developing complex interventions in health it is important to construct a model of the intervention, based on existing data and similar interventions and an idea of the theory behind the proposed interventions as the first step (Richards, 2015).

The development work for the education model started in June 2014 and ended in April 2017. The model is based on a review of literature, a previous study (Pettersson *et al*., 2018) investigating knowledge about diabetes, and experiences from previous research on beliefs about health and illness (Hjelm *et al*., 1999; 2003; 2005; 2013). It was developed in collaboration between researchers experienced in diabetes care and migration and health, and staff working in a multi-professional diabetes team. The diabetes team worked at a diabetes clinic in a primary health care centre in an area of Sweden where a fifth of the population was born abroad (Statistiska Centralbyrån, 2018).

Literature was searched in PubMed, Cinahl and Cochrane Library, as well as governmental reports from the National Board of Health and Welfare, the Swedish Agency for Health Technology Assessment and Assessment of Social Services and national guidelines for diabetes care, particularly in the following areas: culturally adapted diabetes education for foreign-born persons diagnosed with type 2 diabetes, development of education models, pedagogical methods and diabetes and diabetes care. References in recently published studies were scrutinised and chain searching by hand was also performed. Discussions were held with front-line researchers regarding key references.

From the previous study (Pettersson *et al*., 2018) knowledge gaps concerning diabetes could be identified. Experiences from qualitative research using focus-group interviews to investigate beliefs about health and illness (Hjelm *et al*., 1999; 2003; 2005; 2013) were drawn on to develop the instrument (an interview guide) for the focus-group discussions in the proposed programme following the research.

Finally, the results were discussed between the researchers and the multi-professional diabetes team, including a diabetes specialist nurse, a general practitioner, a podiatrist and a dietician, working in a migrant-dense area in Sweden. All those involved had expertise in diabetes education and patient education. Clinical experiences could thus be taken into consideration and reflected upon in a theoretical framework.

## Diabetes education model for migrants with type 2 diabetes

In summary, this education model is planned to be culturally appropriate diabetes education conducted in focus-group discussions proceeding from the person’s individual beliefs about health and illness based on the person’s knowledge. The diabetes education should be held at a primary health care centre and implemented by a multi-professional diabetes team, led by a nurse specialised in diabetes care, assisted by an interpreter. The focus-group discussions are guided by a thematic semi-structured interview guide. The model is planned to include five sessions, each one approximately 90 min long, and the education should be completed in three months. The evidence for this is presented in the text below and the model is described in Table 1.

**Table 1.** Overview of the sessions in the diabetes education model

Session	1	2	3	4	5
Theme for discussion, content	Individual beliefs about illness; why they got diabetes, how it was discovered, how it might affect the body, normal glucose uptake, and beliefs about future health and fears related to diabetes.	Individual beliefs about how to achieve good glycemic control to maintain health and how to recognise changes in blood glucose. Self-care measures and medications focussed.	Individual beliefs about why diabetes-related short- and long-term complications occur, how they affect the body and how they can be prevented to promote and maintain health. Focus on daily foot care and prevention of ‘the diabetic foot’.	Individual beliefs about diet and eating habits and basic principles for dietary adjustment to achieve good glycemic control.	Summarising individual beliefs about how to achieve good glycemic control through the complex relation between diet, exercise, medications and awareness/knowledge and answering participants’ questions.
Staff attending	Diabetes specialist nurse, leader of the session	Diabetes specialist nurse, leader of the session	Diabetes specialist nurse, leader of the session	Diabetes specialist nurse, leader of the session	Diabetes specialist nurse, leader of the session
	Interpreter	Interpreter	Interpreter	Interpreter	Interpreter
		Physician – GP^a^		Dietician	Dietician
					Physician – GP^a^
Venue	Diabetes clinic in a primary healthcare centre				
Teaching method	Focus-group interviews, to reach individual beliefs about health and illness, based on knowledge, determining health-related behaviour. A thematic interview guide, with open-ended questions and critical situations/health problems described, to facilitate interaction/discussions. Knowledge gaps or irrelevant beliefs disclosed to be discussed and content related to main principles for diabetes care.				
Group size	4–5 participants/group				
Length of session	About 90 min/session				
Time planning		Two weeks after first session	Four weeks after first session	Eight weeks after first session	Twelve weeks after first session
Total duration					About three months

a GP – General practitioner.

### The culturally appropriate education diabetes model based on the person’s individual beliefs about health and illness and the person’s knowledge

**Figure 1.**
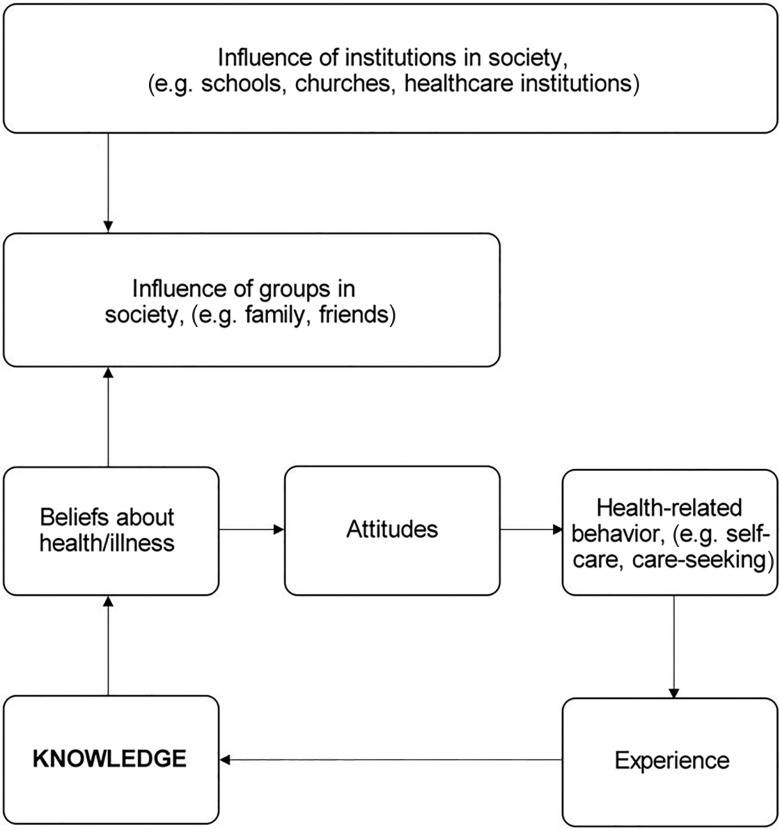
Theoretical framework of the education model. Health-related behavior, including self-care, is determined by beliefs about health and illness held by the individual, based on the person’s knowledge and refined by experiences. Individual beliefs are culturally determined, transmitted through language and learned by socialisation in contact with others in the family, other groups and institutions in the society (e.g. schools, churches, health care institutions).

The theoretical framework for this model is that culturally appropriate diabetes education is defined as education that takes into account cultural and religious perceptions as well as language, providing information to same-gender groups and also adapting food advice to suit the particular community (Attridge *et al*., 2014). Thus, the model is planned to be tailored to the patients’ beliefs and understanding and aimed to develop awareness of the seriousness of the condition in order to influence self-care behaviour (Hjelm *et al*., 1999; 2003; 2005; 2013). Individual beliefs about health and illness are based on knowledge, and constitute attitudes towards health-related behaviour, including self-care, which they guide (see Figure 1) (Hjelm *et al*., 1999; 2003; 2005; 2013; Glanz *et al*., 2008). The health belief model (HBM; Rosenstock *et al*., 1988), including perceived threat, seriousness of and susceptibility to a disease, and degree of self-efﬁcacy (Bandura, 1995), can explain health-related behaviour. A high level of self-efficacy (confidence) contributes to active behaviour and has been shown to be increased by knowledge gained from structured diabetes education in groups (Chatterjee *et al*., 2018; Miller *et al.*, 2020). This leads to better health outcomes and well-being.

Individual beliefs are culturally determined, transmitted through language and learned by socialisation and contact with others in the family, other groups and institutions in society (e.g. schools, churches, health care institutions) (Berger and Luckmann, 1991). Thus the health care education should be individualised, taking account of cultural differences. The advice and recommendations given should, as far as possible, be adapted to the patient’s individual beliefs, needs and wishes (Hjelm *et al*., 1999; 2003; 2005; 2013) as stated in the laws guiding healthcare (SFS, 2014; 2017). Health education, according to educational theory, should be implemented in a learner-centred manner respecting cultural, social and religious values to have the greatest impact (Rogers and Freiberg, 1994; Knight *et al*., 2006). The role of the healthcare staff is to facilitate learning by eliciting the person’s individual beliefs, stimulating interaction/discussions and supporting with information if needed. The participant (patient) is the expert on their health and should be in the centre.

In summary, previous systematic reviews (Hawthorne *et al*., 2008; Attridge *et al*., 2014; Creamer *et al*., 2016) found that culturally appropriate diabetes education showed consistent benefits over conventional care in terms of glycaemic control and diabetes knowledge, sustained in the short to mid-term.

### Educational method

To reach the participants’ individual beliefs, a teaching strategy based on focus-group interviewing technique was chosen (Krueger and Casey, 2015). Guided by a skilled interviewer, the participants share their ideas, beliefs and experiences, influencing each other by responding to ideas and comments in the discussions. Through the interaction in the groups, the participants’ thoughts will get carried away by the discussions and thus more or less conscious beliefs will be revealed (Tang and Davis, 1995; Krueger and Casey, 2015).

The aim of focus groups is to listen and gather information and opinions from participants (Krueger and Casey, 2015). The method is particularly useful for exploring people’s knowledge and experiences about a subject and investigating why they think in a certain way and how they act (Kitzinger, 1995). Here the aim is: (a) to better understand patients’ own beliefs about health and illness; (b) to understand how the respondents feel or think about diabetes and diabetes care; (c) to facilitate learning by using the group interaction and (d) to let the participants share their experiences of living with the chronic disease in order to learn to cope with it (Hjelm *et al*., 2003; 2005; 2013; Krueger and Casey, 2015).

During the different sessions, the participants are asked to discuss experiences of certain themes or in relation to critical situations/health problems described in the respective sessions concerning diabetes and self-care. The participants respond to open-ended questions, posed by a moderator, the nurse specialised in diabetes care, who leads the discussion in the group. The focus for the nurse and moderator is to create a welcoming environment for the participants that encourages them to share their understanding and experiences, and promote an equal environment without letting any participant become dominant in the group (Krueger and Casey, 2015). In addition to the nurse, an interpreter should be present at all sessions, and at each session one of the diabetes team members – the nurse, the podiatrist, the physician, or the dietician – will attend, linked to their particular area of competence. At the last session all members of the diabetes team participate.

To facilitate the discussions, an interview guide is used, with broad questions and descriptions of critical situations/health problems, encouraging discussions of individual beliefs. The areas in the interview guide and thus the themes for the different sessions are presented in Table 1.

Previous research has found that education models should be tailored to the patient’s individual beliefs and aimed to develop awareness of the seriousness of the disease (Hjelm *et al*., 2003; 2005). When needed healthcare staff present at the sessions answer questions raised during the discussions, explain certain issues to the participants and ensure that basic principles of diabetes care are covered. Thus, this education is individualised and person-centred, as recommended in the laws regulating healthcare in Sweden (SFS, 2014; 2017).

### Implementation

The model is planned to include five sessions, each one approximately 90 min long, and the education should be completed in three months and be held at a primary health care centre. Interventions over three months have shown better outcomes than shorter ones (Attridge *et al*., 2014; Creamer *et al.*, 2016). The longest improvement over time is seen in using group sessions for education only (Creamer *et al.*, 2016). The amount of contact time to give significant effect has not been analysed, but five sessions seemed to be reasonable judging from previous studies (Hawthorne *et al*., 2008; Attridge *et al*., 2014;Creamer *et al.*, 2016). Experiences from studies researching individual beliefs about health and illness in focus groups, the chosen educational strategy (Hjelm *et al*., 1999; 2003; 2005; 2013), support the number and length of sessions. Management of persons with type 2 diabetes, including diabetes education, is recommended to be held in primary health care (Socialstyrelsen, 2018; ADA, 2019) and with few exceptions previous interventions have been implemented in the community (Hawthorne *et al*., 2008; Attridge *et al*., 2014, Creamer *et al*., 2016).

When planning the sessions it is important that participants are selected with a view to homogeneity, so that they will feel free to share their feelings and opinions within the group (Krueger and Casey, 2015). In this model the selection criteria are: being diagnosed with and living with type 2 diabetes and practising self-management of the disease. Further, the participants should have a similar background as regards country of birth/ethnic origin and language, age and gender, as culturally appropriate health education should deliver information to same-gender groups and adapted to a particular community (Socialstyrelsen, 2012; Attridge *et al*., 2014).

When complex issues or sensitive topics are discussed, the focus groups should be small, with three to five participants, because small groups are easier to host and run and are comfortable for the participants (Tang and Davis, 1995; Côté-Arsenault and Morrison-Beedy, 1999; Krueger and Casey, 2015). Using a small-group design allows all the members to tell their stories in full (Choi and Rush, 2012) and enables them to learn about their health behaviours (Krueger and Casey, 2015). It is also important to be aware of language barriers in diabetes education for migrants (Choi and Rush, 2012), and therefore an interpreter should be present. This is another reason for limiting the group size. The interaction in the group is more important than the number of participants (Tang and Davis, 1995), and the moderator is the one responsible for facilitating the process of interaction (Krueger and Casey, 2015). In reality this means ensuring that all participants are involved in the discussions, and it prevents anyone from taking over. The choice of the small-group design is also supported by experiences from previous studies investigating beliefs about health and illness in migrants in the presence of an interpreter (Hjelm *et al*., 1999; 2003; 2005; 2013).

In this group education model the participants are recruited by telephone, by a nurse specialised in diabetes care. The interpreter, if needed, should be an authorised professional, meaning a qualified and trained person specialised in healthcare, and working in a face-to-face encounter, as recommended (Hadziabdic and Hjelm, 2013). The interpreter should be recruited from an interpreter agency and should have the competence, language/dialect, ethnic origin, religious background, gender, social group, appearance and attitude required, for the participants’ needs (Hadziabdic and Hjelm, 2013).

The sessions are performed in a secluded room at a health care clinic, as the venue should be a familiar environment that is socially acceptable to the participants (Krueger and Casey, 2015). Sessions should be relaxed, with comfortable seating and some refreshments. The participants should sit in a circular arrangement with an open space in front of them to promote the interaction between them.

A particular aim of the sessions is to support the participants in developing an understanding of the complex relationship between blood glucose control and management in terms of diet, exercise and self-management, in order to promote health and prevent complications related to diabetes. Learning to be observant to changes in blood glucose and the relation between symptoms, pathophysiology and actions is crucial, and therefore knowledge from different subjects, particularly medical science and nursing science, should be integrated.

### Multi-professional diabetes team

The educational model includes a multi-professional diabetes team – a physician, a podiatrist, and a dietician, led by a diabetes specialist nurse (DSN) – as diabetes is a complex disease that needs to be understood and managed in a holistic way, including knowledge from different professions and subjects (Bodenheimer *et al*., 2002; Socialstyrelsen, 2018; ADA, 2019). The DSN has specialist competence in the subject of diabetes and diabetes care, and is also particularly trained and skilled in person-centred diabetes education (Socialstyrelsen, 2018). The DSN should attend all sessions as it has previously been shown to be valuable to have one person who is always present, contributing to the sense of continuity and thus security among the participants, which has a positive influence on glycaemic control (Attridge *et al*., 2014). A positive relation has also been found between care delivered by a DSN and glycaemic control.

### Framework for content

The themes for discussion at the five sessions in this educational model are based on: (1) the Swedish national guidelines for diabetes care (Socialstyrelsen, 2018); (2) Swedish law (SFS, 2017), specifically the Health and Medical Services Act; (3) systematic literature reviews about diabetes care for migrants and ethnic minority groups (Attridge *et al*., 2014; Joo, 2014; Creamer *et al*., 2016); (4) literature about focus-group discussions (Kitzinger, 1995; Krueger and Casey, 2015); (5) nursing self-care theory (Orem, 2001); (6) previous research on individual beliefs about health and illness (Hjelm *et al*., 1999; 2003; 2005; 2013) and (7) a previous study showing knowledge deficits concerning diabetes and diabetes care (Pettersson *et al*., 2018).

The sessions (see Table 1) follow a thematic interview guide with open-ended questions. The first session is based on the participants’ own individual beliefs about illness and their own thoughts about why they contracted type 2 diabetes. In addition, the participants discuss how the disease was discovered, how it might affect the body and normal glucose uptake. Finally, beliefs about future health are also discussed: how long it will persist and fears related to DM.

In the second session, the participants discuss their individual beliefs about how to achieve good glycaemic control, including the four cornerstones: food, exercise, medication and awareness/knowledge. They also learn how to recognise changes in blood glucose control and discuss self-care measures, including the use of nature cure remedies or alternative medicine, in relation to critical situations/health problems with changed control (e.g. infections, hyper- or hypoglycaemia). A physician should also attend this session. At the third session the participants discuss their individual beliefs about why diabetes-related short- and long-term complications occur, how they affect the body and particularly how they can be prevented. A podiatrist should also attend this session, with whom the participants have the opportunity to discuss daily foot care and how to prevent ‘the diabetic foot’. During the fourth session, diet and eating habits are discussed, and the participants discuss their individual beliefs about how they should eat in relation to type 2 diabetes, thus, principles for dietary adjustment. A dietician should also attend the session. The fifth and last session is planned to summarise beliefs about how to achieve good glycaemic control and the complex relation between diet, exercise, medication and awareness/knowledge. Thus the effects of exercise, diet and medication are also discussed, and how to assess blood glucose control. At this session, the whole diabetes team is present, in order to contribute to a holistic picture of the management and to answer any of the participants’ questions. In addition, during the sessions, booklets are handed out in the participants’ native language, concerning diabetes-related complications and self-care.

## Discussion

The content of this article is unique since it describes a culturally appropriate health diabetes education model based on individual beliefs about health and illness and knowledge conducted in focus-group discussions guided by a semi-structured interview guide in five sessions, led by a diabetes specialist nurse in collaboration with a multi-professional team, and completed in three months.

Despite a previously expressed need, culturally tailored diabetes education models for migrants are lacking (Hawthorne *et al*., 2008; Attridge *et al*., 2014; Creamer *et al*., 2016) or have not been evaluated (Chatterjee *et al*., 2018). This is why the model developed here is important and aimed to fill a knowledge gap. The present model differs from previous attempts (Hawthorne *et al*., 2008; Attridge *et al*., 2014; Creamer *et al*., 2016) as it starts from the participants’ own beliefs about health and illness, based on their knowledge. Because beliefs are culturally determined and learned by socialisation (Berger and Luckmann, 1991), the model is culturally tailored and person-centred, delivered by a multi-professional team instead of having education sessions consisting of structured lectures, where the educator, usually a healthcare professional, teaches the patient about diabetes care. Previous research has found that group-based education resulted in improvements in patients’ knowledge about diabetes and glycaemic control (Hawthorne *et al*., 2008; SBU, 2009; Steinsbekk *et al*., 2012; Attridge *et al*., 2014; Creamer *et al*., 2016; Chatterjee *et al*., 2018; Miller *et al*., 2020). National guidelines for diabetes care (Socialstyrelsen, 2018) and evaluations of diabetes care (SBU, 2009; Socialstyrelsen, 2012) likewise recommend group-based education for persons with type 2 diabetes, and therefore focus-group discussions were chosen as the teaching strategy.

The interaction between the participants not only facilitates exchange of beliefs and knowledge (Krueger and Casey, 2015) but group-based education has also been found to be positive for perceived social support, which has shown positive effects on self-care in persons with type 2 diabetes (Berterö and Hjelm, 2010).

A dietician participates during one session together with the nurse specialised in diabetes care, and can give advice if needed based on participants’ own eating habits linked to dietary recommendations in the national guidelines for diabetes care (Socialstyrelsen, 2018). In previous studies (Attridge *et al*., 2014) the cultural dietary preferences and eating habits of the target population have been discussed, based on traditional food in the participants’ country of origin. This, which is not always relevant for the participants, since habits, like eating habits, may change in the new country during the acculturation process (Berry, 2005).

Beliefs about health and illness, including lay beliefs about causes of diabetes, are important cultural aspects that should be taken into account in culturally tailored education (Attridge *et al*., 2014). This education model is based on participants’ individual beliefs about health and illness irrespective of their religion or country of origin, compared to previous studies where cultural beliefs, cultural misconceptions and myths regarding diabetes were discussed based on the participants’ country of origin (Attridge *et al*., 2014). It is of great importance to remember that cultural representations and generalisations, such as beliefs about health and illness, can vary within the same ethnic group and thus need to be assessed individually (Hjelm *et al*., 1999; 2003; 2005).

The present model for group-based education, implemented with focus-group interviews as a teaching strategy is new, and the size of the groups is smaller than recommended for focus groups (three to five participants instead of six to eight) (Krueger and Casey, 2015). However, considering critical factors for the focus-group interview method, such as the use of an interpreter (Hadziabdic and Hjelm, 2013) and the complexity of the topic, a small group is preferable (Tang and Davis, 1995; Côté-Arsenault and Morrison-Beedy, 1999; Krueger and Casey, 2015). The most important thing is to promote interaction between the participants and achieve good discussions, which is easier in a small group design (Krueger and Casey, 2015). The interaction in the groups is more important than the size of the group (Tang and Davis, 1995).

This education model is led by a nurse specialised in diabetes care who acts as a moderator, promoting interaction and discussions between the participants in the group. The diabetes specialist nurse provides support with new knowledge when knowledge gaps are found. This means that, by focussing on the participants’ individual beliefs and knowledge about health and illness, that is, internal resources, the diabetes specialist nurse will use the educative and supportive nursing system in facilitating learning about diabetes and self-management, and thereby improve a person’s self-agency (Orem, 2001). Further, individual members of the multi-professional team attend during the five different sessions and finally in the last session all of them attend to be able to cover different areas of diabetes management and respond to the participants’ questions. Multi-professional diabetes teams involved in education have been found to contribute to significant improvement in glycaemic control and knowledge about diabetes and are therefore recommended (Attridge *et al*., 2014: Creamer *et al*., 2016).

The education model described here has been developed to achieve the goal of a culturally appropriate programme, which is to develop culturally and linguistically appropriate services that function within the context of the cultural beliefs, behaviour and needs presented by an ethnic group (Attridge *et al*., 2014). Since each culture has distinct values and beliefs concerning health, health behaviours and eating practices, culturally appropriate education programmes are necessary to help individuals adopt dietary modifications relevant to their cultural preferences (Auslander *et al*., 2002). But most importantly, this education is based on the participants’ individual beliefs about health and illness, determined by cultural background (Hjelm *et al*., 2003; 2005; 2013), and planned to improve knowledge about type 2 diabetes and thus increase self-care and improve health.

## Conclusion

It has been found that migrants have difficulties assimilating knowledge about diabetes in existing education models offered by health care. Therefore a culturally appropriate diabetes education model has been developed, conducted in a focus-group discussion based on the participants’ individual beliefs about health and illness and their knowledge about diabetes and diabetes care and experiences of self-care. The education model is therefore both individually and culturally tailored and intended to improve knowledge about type 2 diabetes among migrants and thus increase self-care behaviour and improve health.

### Strengths and limitations

It is a strength that many aspects, from both a scientific and clinical perspective, and a theoretical base were considered when this education model was developed in collaboration with a multi-professional diabetes team. Further, this education model ought to be appropriate for several population groups considering that it is based on the participants’ individual beliefs about health and illness and can be implemented at different phases during the illness trajectory and thus be delivered at various times. However, one limitation might be that the model is not tested yet, which is the next step in the research process. Then an evaluation from the users’ perspective will also be included to further revise the model.
